# Views of patients with obesity on person‐centred care: A Q‐methodology study

**DOI:** 10.1111/hex.13609

**Published:** 2022-09-30

**Authors:** Paige I. Crompvoets, Jane M. Cramm, Elisabeth F. C. van Rossum, Anna P. Nieboer

**Affiliations:** ^1^ Department of Socio‐Medical Sciences, Erasmus School of Health Policy and Management Erasmus University Rotterdam Rotterdam The Netherlands; ^2^ Department of Internal Medicine, Division of Endocrinology, Erasmus MC University Medical Center Rotterdam Rotterdam The Netherlands; ^3^ Obesity Center CGG, Erasmus MC University Medical Center Rotterdam Rotterdam The Netherlands

**Keywords:** care provision, obesity, patient views, person‐centred care, Q methodology

## Abstract

**Introduction:**

To better accommodate patients with obesity, the adoption of a person‐centred approach to healthcare seems to be imperative. Eight dimensions are important for person‐centred care (PCC): respect for patients' preferences, physical comfort, the coordination of care, emotional support, access to care, the continuity of care, the provision of information and education, and the involvement of family and friends. The aim of this study was to explore the views of patients with obesity on the relative importance of the dimensions of PCC.

**Methods:**

Q methodology was used to study the viewpoints of 21 patients with obesity on PCC. Respondents were asked to rank 31 statements about the eight dimensions of PCC by level of personal significance. Using by‐person factor analysis, distinct viewpoints were identified. Respondents' comments made while ranking were used to verify and refine the interpretation of the viewpoints.

**Results:**

Five distinct viewpoints were identified: (1) ‘someone who listens in an unbiased manner’, (2) ‘everything should run smoothly’, (3) ‘interpersonal communication is key’, (4) ‘I want my independence’, and (5) ‘support for myself and my loved ones’. Viewpoint 1 was supported by the largest number of respondents and explained the most variance in the data, followed by viewpoint 3 and the other viewpoints, respectively.

**Conclusion:**

Our findings highlight the need for tailored care in obesity treatment and shed light on aspects of care and support that are most important for patients with obesity.

**Patient Contribution:**

Our sample consisted of patients. Patients were also involved in the development of the statement set through pilot testing.

## 1 INTRODUCTION

Over the past four decades, the global prevalence of obesity has nearly tripled.^1^, ^2^ The World Health Organization defines obesity as an excessive accumulation of body fat that poses a threat to health.^3^ Living with obesity seriously impairs physical and psychosocial functioning, resulting in a reduced quality of life.^4^ Obesity also increases the risk of developing other serious health conditions, such as type 2 diabetes, cardiovascular diseases, several types of cancer, and many other diseases.^5^ Consequently, obesity, and especially severe obesity is associated with increases in healthcare utilization and expenditures, as well as substantial societal costs due to productivity losses.^6^, ^7^ Although many health institutions have recognized it as a chronic disease,^8^ healthcare systems seem poorly prepared to meet the needs of patients living with obesity. Clinical guidelines for the treatment of these patients are often too simplistic, focusing merely on weight loss instead of the improvement of overall health and well‐being.^9^ As a result, individual circumstances, including contributing factors and underlying diseases, are often overlooked.^10^ Furthermore, patients with obesity often experience weight‐related stigma and discrimination in healthcare, which can affect the quality of their care and their treatment outcomes.^11^, ^12^ For instance, some healthcare professionals view patients with obesity more negatively than other patients and spend less time treating them.^13^ Healthcare professionals may also be insufficiently equipped or educated to perform standard medical procedures on patients with obesity.^14^

To better accommodate patients with obesity, the adoption of a person‐centred approach in which care is tailored to the individual and individuals' preferences, needs, and values are respected seems to be imperative.^15^ Person‐centred care (PCC) can be seen as a paradigm shift in healthcare that has been gaining broad support with the increasing interest in the quality of care.^16^, ^17^ The Picker Institute distinguishes eight dimensions that are important for PCC: respect for patients' preferences, physical comfort, coordination of care, emotional support, access to care, continuity of care, the provision of information and education, and the involvement of family and friends.^18^, ^19^ An overview of these dimensions can be found in Table 1.

**Table 1:** The eight dimensions of PCC

Patients' preferences	Treating patients with dignity and respect and demonstrating sensitivity to their preferences, needs and values. When treating patients with obesity, a focus on overall quality of life, rather than the achievement of weight loss alone, is important.^20^
Physical comfort	Physical comfort should be supported, in the case of obesity by offering pain management if needed and attending to problems with physical activity. Buildings should be comfortable and provide enough privacy. Specifically, a lack of privacy during weight assessment has been identified as a barrier to the engagement in care of some patients with obesity.^21^
Coordination of care	Coordination and integration of care among healthcare professionals within organizations is critical. All professionals should be well informed, and each patient should have a primary contact person.
Emotional support	Living with obesity is associated with a great psychosocial burden, and patients with obesity may experience issues such as depression, anxiety, stigma and discrimination.^22^
Access to care	Includes quick and easy appointment scheduling, accessible buildings and access to adequate medical equipment. Not all currently used medical equipment is designed to accommodate patients with larger bodies, which may restrict quality of care and contribute to stigmatization of patients with obesity.^23^
Continuity of care	Includes smooth transitions between healthcare providers and the transferring of relevant patient information between organizations. As patients with obesity often deal with comorbid conditions, several providers in primary and specialty care settings may be involved in their care.^24^
Information and education	Patients should receive appropriate information and education about all aspects of their care. Accumulating evidence links low health literacy to excess body weight.^25^ To support patients with obesity to be in charge of their own care, the provision of understandable information and education is essential.
Family and friends	The involvement of family and friends may also play an important part in caring for patients with obesity, as family members and friends may act as caregivers or contributors to the disease. When applicable, PCC also involves paying attention to the roles of loved ones in obesity treatment.

Abbreviation: PCC, person‐centred care.

PCC has been associated with improved patient outcomes in various healthcare settings,^26^ including the provision of care to patients with obesity.^27^ However, the relative importance of the different aspects of PCC seems to vary among patient groups.^28^, ^29^ Although aspects of care that may be important specifically for patients with obesity have been identified, the significance of the eight dimensions of PCC for patients with obesity has not been assessed. Gaining insight into the aspects of PCC that are most important to this patient group is a vital step toward improved care provision, and consequently improved quality of care and patient outcomes. Thus, the aim of this study was to explore the views of patients with obesity on the relative importance of the dimensions of PCC.

## 2 METHODS

### 2.1 Q methodology

To examine the views of patients with obesity on what is important for PCC, the mixed‐method Q methodology was used. Q methodology may be best described as an inverted factor analytic technique for the systematic study of subjective viewpoints.^30^ Q‐methodology research aims to identify and discern views on a specific topic, rather than determine the prevalence of these viewpoints. In a Q‐methodology study, respondents are asked to rank a set of statements about the study subject. Using by‐person factor analysis, in which the respondents are treated as variates, distinct viewpoints are identified. Q methodology has been used to examine the views of patients and professionals, such as patients with multimorbidity,^28^ those with end‐stage renal disease,^29^ and professionals and volunteers providing palliative care,^31^ on what is important for PCC.

### 2.2 Respondents

As our goal was to obtain a wide breadth of views on what is important for PCC for patients with obesity, we recruited respondents varying in terms of gender, age, educational background, marital status, and health literacy. Eligible patients were over the age of 18 years and had body mass indices (BMIs) of at least 40 kg/m^2^, which defines severe obesity. This obesity threshold was chosen because it is associated with the most healthcare utilization and greatest health risks.^5^, ^6^ Practitioners working in the internal medicine departments of four hospitals in the area of Rotterdam, the Netherlands, informed patients about the study. In the Netherlands, access to nonurgent hospitals or specialty care requires a referral from a general practitioner (GP).^32^ Recruitment through hospitals thus ensured that respondents were familiar with both specialty and primary care (e.g., GP visitation), characteristic of care provision for patients with severe obesity.^6^, ^24^ Data collection took place between April and October 2021. Twenty‐six eligible patients gave consent to be contacted to receive detailed study information and schedule an appointment. Of the 26 patients that were contacted, 3 were unable to schedule appointments and 2 could not be reached by the researcher. This led to the inclusion of 21 patients in the study, which is a typical sample size for a Q‐methodology study.^30^ Q‐methodological research requires only a small number of purposively selected respondents to represent the breadth of views in a population.^30^ Consultation of the literature and the expert opinion of a professor of obesity and stress research who is involved in the treatment of patients with obesity revealed no evidence of missing viewpoints.

### 2.3 Statements

To capture the full range of possible views on a specific topic, the statements in a Q‐methodology study should have good coverage of the subject of interest.^30^ The eight dimensions of PCC provided by the Picker Institute were used as a conceptual framework for this study.^18^, ^19^ First, statements from previous studies in which the same framework was used to investigate the views of patients or professionals on what is important for PCC were collected.^28^, ^29^, ^31^ Further statement selection was informed by various sources covering the care and support needs of patients with obesity, such as scientific articles^23^, ^33^ and clinical guidelines,^34^ as well as the autobiographies and social media posts of individuals living with obesity. In an iterative process, all members of the research team, including an internist‐endocrinologist who is a professor in the field of obesity and stress research and involved in clinical care provided to patients with obesity, generated, reviewed, and revised statements. A final set of 31 statements was constructed and pilot tested with three respondents fulfilling our inclusion criteria. Based on the pilot testing results, a few adjustments to the phrasing of some statements were made (see Supporting Information: Appendix A). No substantive change was required, and no missing statement was revealed. The final statement set is provided in Table 2. Because no substantial change was made to the statement set, the pilot data were included in the analyses conducted for this study.

**Table 2:** Statements and factor arrays

		Factor/viewpoint^a^				
#	Statement	1	2	3	4	5
	*Patient preferences*					
1	Being treated with dignity and respect	+4	+1	+2	+2	+4
2	Unbiased healthcare professionals	+3	0	+1	−1	−3
3	A focus on my quality of life	0	−2	−2	+3	−2
4	Being involved in decisions	+3	+2	0	0	+3
5	Taking into account my preferences	+1	−4	−3	−1	+1
6	A focus on what I can do myself	−1	−1	0	+2	−2
	*Physical comfort*					
7	Attention to my physical comfort	+1	0	−2	+2	0
8	Attention to problems with physical activity	0	+3	−1	+4	−2
9	Comfortable waiting area and treatment rooms	−3	−3	−4	−3	+1
10	Sufficient privacy in the waiting area and treatment rooms	−2	−3	−1	−4	0
	*Coordination of care*					
11	Well‐informed healthcare professionals	+2	+2	+2	0	−1
12	Practitioners who coordinate care and advice properly	+2	+4	+4	0	−2
13	Knowing where to go with questions	−2	0	0	+1	−3
	*Emotional support*					
14	Healthcare professionals who really listen to me	+4	0	+2	0	0
15	Attention to my emotions	+2	+1	−4	−3	−1
16	Attention to the influence of my health on my life	+3	+1	−2	+2	−1
	*Access to care*					
17	Available and accessible healthcare	−2	+3	−1	+1	−1
18	Sufficient time during appointments	+1	‐4	+4	+1	+2
19	Availability of appropriate resources and facilities	0	−2	+1	−2	+2
20	That money is not a problem	−1	0	+2	0	−4
21	Being able to schedule an appointment quickly and easily	−1	−1	−3	+1	−4
	*Continuity of care*					
22	Being well informed during a referral	0	+2	+3	−3	−1
23	That my information is transferred properly with a referral	+1	+4	+1	−2	+3
24	Knowing where to go for care and support after treatment	−3	+1	+1	+4	+2
	*Information and education*					
25	Being well informed about all aspects of my care	+2	−2	+3	+3	+4
26	Easy access to my own medical data	−2	−2	0	+2	+2
27	A good explanation with all information	0	+2	+3	−2	0
28	Assistance with healthy living	−1	+2	0	−2	−4
	*Family and friends*					
29	That my loved ones can participate in the decision‐making	−4	−2	−3	−1	+1
30	Attention to questions and needs of my loved ones	−3	−2	−2	−1	0
31	Help from healthcare professionals to get support from my loved ones	−4	−3	−1	−4	+3

^a^ Viewpoints: 1, ‘someone who listens in an unbiased manner’; 2, ‘everything should run smoothly’; 3, ‘interpersonal communication is key’; 4, ‘I want my independence’; 5, ‘support for myself and my loved ones’.

### 2.4 Data collection

**Figure 1:**
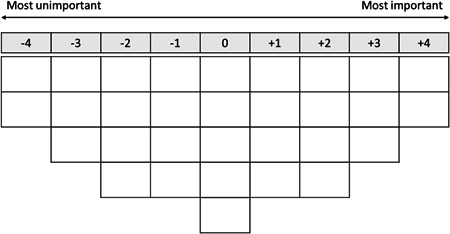
Sorting grid.

Data collection took place in an online environment using video conferencing software; the process lasted approximately 60 min per respondent. One researcher guided the respondents' ranking of statements. All sessions were audio recorded with respondents' informed consent. First, the respondents answered basic demographic questions and filled in the Set of Brief Screening Questions (SBSQ) as an assessment of health literacy.^35^ Low health literacy was defined as an average SBSQ score of 2 or lower. Next, the respondents were asked to carefully read the statements about aspects of PCC, displayed on the screen one by one in random order using the HtmlQ software,^36^ and to sort them into ‘important’, ‘neutral’, and ‘unimportant’ piles. The researcher then asked the respondents to rank the statements in each pile according to their personal significance using a forced sorting grid with a scale ranging from +4 (*most important*) to –4 (*most unimportant*; Figure 1). While ranking, the respondents were encouraged to speak out loud about their views; after completing the ranking, they were asked to elaborate on their placement of the statements. All comments made by the respondents during and after the ranking process were transcribed verbatim.

### 2.5 Statistical analysis

To identify distinct viewpoints on what is important for PCC for patients with obesity, the rankings of the 21 respondents were intercorrelated and subjected to by‐person factor analysis using the PQMethod software.^37^ Clusters in the data were identified using centroid factor extraction and varimax rotation. Potential factor solutions were evaluated by considering the total of associated respondents at a significance level of 0.05 (i.e., a factor loading of ±0.42), upholding a minimum of two associated respondents per factor, and the percentage of explained variance. Fulfilment of the Kaiser–Guttman criterion, which suggests that only factors with eigenvalues of 1.0 or more be retained, was examined.^38^, ^39^ To finalize our decision on the number of factors to retain, qualitative data (i.e., comments made by the respondents during and after ranking) were considered. For each factor or viewpoint, the rankings of associated respondents were merged by calculating weighted averages, thereby forming a ‘factor array’ that depicted how a typical respondent holding that viewpoint would rank the statements. As our aim was to gain a broad understanding of respondents' diverse viewpoints, our interpretation was based on these factor arrays. For each viewpoint, statements ranked as most important (+3 and +4) and most unimportant (–3 and –4) and distinguishing statements (ranked significantly higher or lower than in other viewpoints) were inspected. The qualitative data were used to verify and refine our interpretation of the viewpoints.

## 3 RESULTS

Twenty‐one respondents completed the ranking (Table 3). The analysis revealed five factors, or distinct viewpoints, that together explained 48% of the variance in the data. Data from 17 (81%) of the 21 respondents were associated significantly with one of the five viewpoints (*p* ≤ .05). Data from two respondents were associated with two viewpoints each, and those from two respondents were not associated significantly with any factor. All viewpoints were supported by at least two respondents; viewpoints 1 and 3 were supported by 7 and 4 respondents, respectively. Viewpoint 5 had an eigenvalue of 0.95, just below the Kaiser–Guttman cut‐off of 1.0, but the qualitative data indicated that it was meaningful and distinguishable from the other viewpoints. The degree of correlation between viewpoints was low to moderate (*r* = –.15 to .37). The factor arrays for the five viewpoints are provided in Table 2.

**Table 3:** Demographic characteristics of respondents (*n* = 21)

Characteristic	*N*	Percentage
Gender (female)	17	81
Age		
20–29	8	38
30–39	4	19
40–49	5	24
50–59	3	14
60–67	1	5
Marital status		
Married	9	43
Single	6	29
Living together with partner	6	29
Education		
Primary school	1	5
Secondary school	5	24
Vocational education	10	48
Higher education	5	24
Health literacy (low)	4	19

### 3.1 Viewpoint 1: ‘someone who listens in an unbiased manner’

Viewpoint 1 accounted for the most explained variance (17%) in this study. The PCC dimensions most characterizing this viewpoint are ‘respect for patients’ preferences’ and ‘emotional support’. Central to this viewpoint was respondents’ desire to be seen and heard like any other patient without obesity. These patients wish to be treated with dignity and respect (statement 1, +4). Respondent 8 stated ‘You just want to be taken seriously. We are all human, that includes people who are overweight’. They often feel misunderstood because healthcare professionals blame all of their health issues on their weight. [‘You fight against a judgment that you cannot get out of. They do not even examine me. Right off the bat they go: “I can refer you for a stomach reduction”’ (Respondent 18)]. To get the care and support that suits their needs, these patients believe that unbiased healthcare professionals (statement 2, +3) who genuinely listen (statement 14, +4) are crucial. Respondent 13 stated ‘That they look further than your weight, that is the most important thing to me. That it is not like everything that is wrong with you is because of your weight’. They want healthcare professionals to provide emotional support and acknowledge the impact of their health problems on their life [statement 16, +3; ‘I have three small children and it is really hard for me to do things with them just because I am overweight’ (Respondent 6)]. They seek recognition for the complexity of their condition. Respondent 8 stated ‘Recognition that obesity is a disease and it should be treated that way is very important’.

To remain in charge of their care, these patients want to be involved in decisions (statement 4, +3), while leaving friends and family members out of the decision‐making process [statement 29, –4; ‘No, I do not think that is important. I decide what I want’ (Respondent 6)]. Respondents holding this viewpoint ranked all statements covering the ‘involvement of friends and family’ dimension as least important.

### 3.2 Viewpoint 2: ‘everything should run smoothly’

Viewpoint 2 accounted for 8% of the explained variance. Patients holding this viewpoint seek well‐coordinated care and advice (statement 12, +4) and the proper transfer of information in case of referral (statement 23, +4). Respondent 3 stated ‘*The doctors have to agree on what is the best option for me*’. Furthermore, they desire easily accessible care with short wait times [statement 17, +3; ‘That it will not be a lengthy process before I can be helped’ (Respondent 16)].

These patients would also like healthcare professionals to consider their physical comfort by attending to problems with physical activity [statement 8, +3; ‘Stairs are very much a no go for me and it is important that they know that’ (Respondent 16)]. However, they consider other aspects of physical comfort, such as waiting areas and treatment rooms that are comfortable (statement 9, –3) or provide enough privacy (statement 10, –3), to be less important. Respondent 16 stated ‘When I weighed 127 kilos at my heaviest, the seats were a bit uncomfortable, but I do not have that problem now’.

In contrast to those holding viewpoint 1, patients holding viewpoint 2 do not mind if care does not align with their own preferences [statement 5, –4; ‘I do not think that your preferences should be taken into account in a hospital or with a doctor because as human beings we can have a lot of preferences that do not really apply’ (Respondent 16)]. They emphasize their own responsibility for getting the care they need [‘Right now in the Netherlands, you get the right care. As a patient, you also need to be somewhat well‐informed yourself’ (Respondent 16)]. They believe that being well prepared avoids the need for lengthy appointments (statement 18, –4). Respondent 3 stated ‘If I have a question, I just ask it. And if I did not understand something or if I forgot something […] I can just call and ask’.

### 3.3 Viewpoint 3: ‘interpersonal communication is key’

Viewpoint 3 accounted for 10% of the explained variance. It focuses on the exchange of information among all involved parties. Patients holding this viewpoint want to know what to expect, and thus value information about all aspects of their care (statement 25, +3), including information about referrals (statement 22, +3), very highly [‘Because I want to know where I stand, what will happen and what is needed’ (Respondent 10)]. They believe that a good explanation is needed to properly understand information (statement 27, +3). Respondent 7 stated ‘I often feel a bit overwhelmed during consultations. That things are being said for which I was not fully prepared. I sometimes think afterwards, “have I understood everything that has been said?”’. These patients believe that having sufficient time during appointments is a prerequisite for the proper exchange of information (statement 18, +4). They often leave consultations feeling poorly because of unanswered questions. [‘You just notice that they are under time pressure, that it should all happen quickly. You hardly have time for questions, so you do not leave with a good feeling’ (Respondent 10)].

Similarly to those holding viewpoint 2, these patients value the coordination of care and advice among practitioners highly (statement 12, +4). They specifically dislike the conflicting of treatment plans with each other [‘It is important that one practitioner also knows what the other practitioner is doing and that it fits together’ (Respondent 7)].

In contrast to those holding viewpoint 1, these patients prefer that care and support are of an informative nature, rather than attending to emotions that they might be experiencing (statement 15, –4). Respondent 1 stated ‘Things like quality of care are much more important to me than people sitting down to listen to emotions or something like that. To me, emotions and scientific correctness often clash’. Similarly to those holding viewpoint 2, they do not mind if care does not align well with their preferences [statement 5, –3; ‘For me it is really about that the care is good and that it is the best, even if I do not prefer it’ (Respondent 1)].

### 3.4 Viewpoint 4: ‘I want my independence’

Viewpoint 4 accounted for 7% of the explained variance. The aim of remaining independent is central to this viewpoint. In contrast to those holding viewpoints 1–3, patients holding viewpoint 4 want to focus on what they can do on their own (statement 6, +2), as they believe that this will preserve their quality of life [statement 3, +3; ‘I think it is important that I can and may continue to do a lot independently’ (Respondent 17)]. In line with this focus, these patients want healthcare professionals to attend to their problems with physical activity (statement 8, +4). Respondent 17 stated ‘I think it is very important to work on this [problems with physical activity] as much as possible and to expand what is possible to do myself’.

Although these respondents seek independence, they value knowing where to go for care and support after treatment highly (statement 24, +4). They are willing to take the lead, provided that they know where they can go for support. Respondent 4 stated ‘That you have a telephone number and that you can call them with questions or if anything is unclear. I find accessibility very important’. To facilitate independence, they also prefer to be well informed about all aspects of their care (statement 25, +3) and appreciate easy access to their own medical data (statement 26, +2). However, these patients do not require a good explanation of all information provided to them (statement 27, −2) as they have no difficulty understanding their medical data [‘I have been walking in and out of hospitals for so long, most of it is self‐evident’ (Respondent 17)].

In contrast to those holding viewpoints 1–3, patients holding viewpoint 4 find other aspects of the ‘continuity of care’, such as being well informed during referrals (statement 22, –3) and the proper transfer of information upon referral (statement 23, –2) to be less important. They do not mind asking questions or re‐sharing information with professionals [‘I can also tell it myself and I can ask for everything I need and I always do that’ (Respondent 4)].

### 3.5 Viewpoint 5: ‘support for myself and my loved ones’

Viewpoint 5 accounted for 5% of the explained variance. This viewpoint is distinguished by an emphasis on the supporting roles of family members and friends. Patients holding this viewpoint seek support from their loved ones and help from healthcare professionals in obtaining it [statement 31, +3; ‘I am married and I want help from my husband because he really knows a lot about me’ (Respondent 20)]. They also value their autonomy highly; they want to be informed about all aspects of their care (statement 25, +4) and involved in decisions (statement 4, +3). Respondent 20 stated ‘I do not like them talking about me behind my back’. Similarly to those with viewpoint 1, patients with viewpoint 5 consider being treated with dignity and respect (statement 1, +4) to be one of the most important aspects of PCC [‘Everyone has the right to be treated with respect and receive proper care’ (Respondent 5)]. They value comfortable waiting areas and treatment rooms (statement 9, +1) more than patients with other viewpoints, as they appreciate their personal space. Respondent 20 stated ‘I do not think it is necessary that they sit right on top of me in treatment rooms’.

Compared with patients with other viewpoints, those with viewpoint 5 consider some aspects of PCC to be out of reach, and thus rank them as less important. For example, they accept that money may be a problem sometimes [statement 20, –4; ‘Money comes, money goes. It just makes some things a little easier, but if you do not have it, you do not have it’ (Respondent 5)] and they believe that receiving treatment only from unbiased healthcare professionals is not realistic [statement 2, –3; ‘It is not realistic because that [stigmatisation from healthcare professionals] happens, whether you like it or not’ (Respondent 5)].

## 4 DISCUSSION

In this study, five distinct views on what is important for PCC for patients with obesity were identified. Patients holding viewpoint 1, ‘someone who listens in an unbiased manner’, want healthcare professionals to look beyond a patient's weight. This viewpoint explained the most variance in the data and was supported by the largest number of respondents. Patients holding viewpoint 2, ‘everything should run smoothly’, seek care that is well coordinated and accessible. Patients holding viewpoint 3, ‘interpersonal communication is key’, prefer care of an informative nature. Patients holding viewpoint 4, ‘I want my independence’, are driven by the desire to remain independent. Finally, patients holding viewpoint 5, ‘support for myself and my loved ones’, seek help to involve their loved ones in their care. Our findings thus show that patients with obesity hold various views on what is most important in care and support. This diversity may be explained by the multifactorial nature of obesity,^10^ which results in different care needs. Our results suggest that we cannot apply a single standard of care to patients with obesity, and reflect the importance of care that is tailored to each individual.

Although views on PCC varied among patients, ‘being treated with dignity and respect' was deemed to be relatively important across viewpoints. This result is not surprising, as obesity is a highly stigmatized condition, and many individuals living with it report having stigmatizing healthcare experiences, such as disrespectful treatment.^40^ Research suggests that higher patient BMIs are associated with lesser physician respect.^41^ Although many respondents in our study reported stigmatizing healthcare experiences, ‘unbiased healthcare professionals' was not unequivocally ranked as important across viewpoints. Patients holding viewpoint 5 even ranked it as one of the least important aspects of PCC, but they explained this judgment as reflecting their belief that weight‐related stigmatization in healthcare is an unsolvable problem. Furthermore, some respondents with other viewpoints related ‘unbiased healthcare professionals’ strongly to ‘treatment with dignity and respect’, and for practical purposes chose to rank the former statement lower. This perspective has also been identified in research on patients’ views on weight stigmatization in healthcare; patients with obesity agreed that a lack of physician respect results from such stigmatization.^42^

Our results further show notable differences in views on the importance of emotional support. Patients with viewpoint 1 value such support highly, viewing it as fundamental for obesity treatment. In contrast, patients with viewpoint 3 do not want practitioners to attend to their emotions, although they acknowledge the emotional impact of their condition. Many individuals with obesity struggle with psychosocial issues, including psychiatric illness, low self‐esteem, reduced quality of life, and the internalization of weight stigmatization.^22^, ^43^ Thus, multidisciplinary obesity treatment often includes a focus on emotional well‐being, which is suggested to have beneficial effects on health.^44^, ^45^ However, patients with some viewpoints prefer a pragmatic approach. These opposing views may pose a dilemma for healthcare professionals aiming to provide high‐quality and holistic care to patients with obesity. Future research may clarify the emotional support needs of patients with obesity and the relationship of emotional support to treatment outcomes.

The involvement of family and friends was considered to be relatively unimportant across viewpoints in this study, except among patients with viewpoint 5, who seem to depend more on social support. Patients with viewpoint 1 strongly oppose the involvement of loved ones and prefer to make decisions individually. This perspective might be explained by the complexity of living with obesity, which only the patient can understand fully. These findings bring to light new questions about the extent to and the manner in which family members and friends should be involved in obesity treatment. Social support has been shown to be beneficial in chronic illness management,^46^ but literature on the involvement of family and friends in adult obesity treatment is inconclusive.

## 5 LIMITATIONS

Several potential limitations of this study should be considered. First, the sample of patients recruited for this study may seem to be small. However, it meets the requirements of Q methodology^30^ and is similar to those of other studies.^28^, ^47^ Furthermore, consultation of the literature revealed no evidence of a missing viewpoint. Additionally, the viewpoints identified in this study were recognized by a professor of obesity and stress research who is involved in the treatment of patients with obesity and indicated that no viewpoint was missing, based on many years of clinical experience. Furthermore, the representation of the male perspective in this sample might be limited due to the male‐to‐female ratio. However, a similar ratio is seen in patients seeking obesity care.^48^ Second, at the start of the data collection period, respondents could only participate online due to COVID‐19 pandemic precautions. Although we later offered the opportunity for face‐to‐face participation, this approach may have led to the underrepresentation of individuals with low health literacy, for whom digitalization can be a barrier to engagement.^49^ However, the views of individuals with low health literacy are represented in this study, as four respondents met this criterion. Finally, our study was conducted in the Netherlands, and the identified viewpoints may not represent the views of patients living in countries with different health systems. For example, because health insurance is mandatory in the Netherlands, every resident has basic access to care. Aspects of the ‘access to care’ dimension may thus be viewed differently in countries without universal healthcare. However, Dutch health insurance does not cover all obesity treatments. For instance, most weight‐reducing medications are not covered.

## 6 CONCLUSION

Five distinct views on what is important for PCC for patients with obesity were identified. The viewpoint ‘someone who listens in an unbiased manner’ was supported by the largest number of respondents. With these findings, we have begun to shed light on the communalities in the views of patients living with obesity on PCC. Our data shows that the views on what care and support should look like for patients with obesity vary, stressing the need for tailored care for patients with obesity. Future research may build on and expand our study's findings by considering additional factors that might influence how patients with obesity view PCC. For instance, experiences of weight stigmatization in healthcare may lead to different perspectives on what is most important in care and support. Furthermore, we explored the views of patients living with severe obesity. Future studies might examine the views of patients living with less severe obesity and explore to what extent their views on PCC differ.

The views that are described in this paper provide valuable insight into the perspective of patients living with obesity on what is most important in care and support. Importantly, this knowledge helps us to understand what PCC provision for patients with obesity might entail and may help organizations arrange care accordingly. For example, some patients may benefit greatly from a high level of emotional support, while others will respond better to care and support that is centred around patient education or self‐management.

## CONFLICT OF INTEREST

The authors declare no conflict of interest.

## Supporting information

Supporting information.

## Data Availability

The data that support the findings of this study are available from the corresponding author, PI Crompvoets, upon reasonable request.
