# Supplementary material for: Views of patients with obesity on person‐centred care: A Q‐methodology study
Source: Health Expect. 2022 Sep 30;25(6):3017–26. doi: 10.1111/hex.13609 (PMC9700190; doi:10.1111/hex.13609)
Supplement: Supplementary file 1 — Supporting information. [file HEX-25--s001.docx]

Appendix A

Table A1

*Adjustments made to the statement set based on pilot testing (*n *= 3)*

| # | Original statement | Adjusted statement | Argumentation |
| --- | --- | --- | --- |
| 2 | Unbiased and unprejudiced healthcare providers | Unbiased healthcare providers | Removing repetitive words to shorten statement |
| 3 | A focus on my overall quality of life | A focus on my quality of life | Removing redundant words to shorten statement |
| 6 | A focus on my skills and competencies | A focus on what I can do myself | Replacement of difficult to understand words to clarify statement |
| 10 | Sufficient privacy | Sufficient privacy in the waiting area and treatment rooms | Specification of context to specify statement |
| 16 | Attention to the impact of my health on my life | Attention to the influence of my health on my life | Replacement of difficult to understand words to clarify statement |
| 17 | Proper availability and accessibility | Available and accessible healthcare | Specification of context to specify statement |
| 18 | Sufficient time | Sufficient time during appointments | Specification of context to specify statement |
| 31 | Help getting support from my loved ones | Help from healthcare providers to get support from my loved ones | Specification of context to specify statement |
